# Genotype Distribution and Migration Patterns of Hepatitis C Virus in Shandong Province, China: Molecular Epidemiology and Phylogenetic Study

**DOI:** 10.2196/60207

**Published:** 2025-08-18

**Authors:** Lin Lin, Guoyong Wang, Lianzheng Hao, Tingbin Yan

**Affiliations:** 1 HIV/AIDS Control and Prevention, Key Laboratory of Infectious Disease Control and Prevention, Shandong Center for Disease Control and Prevention, Jinan, China; 2 Department of Orthopedic Surgery, School of Basic Medical Sciences, Qilu Hospital of Shandong University, No. 107 Wenhua West Road, Jinan, Shandong Province, 250012, China, 86 531-82169562

**Keywords:** hepatitis C virus, genotypic diversity, phylogenetic analysis, migration patterns, Shandong Province, Bayesian skyline plot, migration, genotype, prevention, disease control, phylogenetic, HCV, genotypic diversity, migration pattern, epidemiological, China

## Abstract

**Background:**

Hepatitis C virus (HCV) remains a significant public health concern in China, particularly in Shandong Province, where detailed molecular epidemiological data are limited. HCV exhibits substantial genetic diversity, and understanding its genotype distribution and transmission dynamics is critical for developing effective control strategies.

**Objective:**

This study aimed to investigate the genetic diversity, geographic dissemination, and evolutionary history of HCV genotypes in Shandong Province, China, using molecular techniques and phylogenetic methods.

**Methods:**

A total of 320 HCV-positive serum samples were collected from multiple hospitals across Shandong Province between 2013 and 2021. HCV RNA was extracted and amplified targeting the 5′ untranslated region (UTR), Core, and NS5B regions. Sequencing was conducted, and genotypes were determined using the National Center for Biotechnology Information’s Basic Local Alignment Search Tool (NCBI BLAST). Phylogenetic trees were constructed using maximum likelihood methods with the general time reversible with Gamma-distributed rate variation among sites [(GTR)+Gamma model]. The temporal and geographic evolution of the major subtypes (1b and 2a) was analyzed using Bayesian Markov chain Monte Carlo (MCMC) methods implemented in Bayesian Evolutionary Analysis Sampling Trees (BEAST). The Bayesian skyline plot (BSP) was used to infer population dynamics and estimate the time to the most recent common ancestor (tMRCA).

**Results:**

Genotypes 1b (n=165) and 2a (n=131) were identified as the predominant subtypes, with a small number of genotypes 3b, 6a, 6k, and potential recombinant strains also detected. Phylogenetic analysis revealed distinct evolutionary clustering of 1b and 2a strains, suggesting multiple diffusion events within the province. The tMRCA of subtypes 1b and 2a were estimated to be 1957 and 1979, respectively. Bayesian skyline analysis showed that both subtypes experienced long-term population stability, followed by a rapid expansion period between 2014 and 2019 (1b) and 2014 to 2016 (2a), respectively. The analysis also identified key transmission hubs such as Jinan, Liaocheng, Tai’an, and Dezhou, indicating city-level variations in HCV spread.

**Conclusions:**

This study provides data-supported insights into the genotypic landscape and evolutionary patterns of HCV in Shandong Province. The identification of dominant subtypes, potential recombinant strains, and regional transmission pathways enhances our understanding of local HCV epidemiology. These findings have implications for public health policy, resource allocation, and targeted treatment strategies. The integration of molecular epidemiology and phylogenetics offers a valuable model for infectious disease surveillance and control in similar settings.

## Introduction

Hepatitis C virus (HCV) is an enveloped virus containing a single-stranded positive RNA genome approximately 9.6 kilobases in length, belonging to the Flaviviridae family, *Hepacivirus* genus [1]. It can lead to acute and chronic hepatitis, and if not treated promptly and effectively, may progress to cirrhosis and hepatocellular carcinoma [2]. HCV infection remains a global health burden, with an estimated 58 million chronic cases, 1.5 million new infections, and 290,000 deaths annually [3]. China is one of the most severely affected countries, with the highest number of HCV infections globally [4].

In recent years, the global epidemic trend of HCV has garnered significant attention [5]. Hepatitis C is primarily transmitted through blood contact and is associated with severe complications, including cirrhosis and liver cancer [6]. The prevalence and distribution of various HCV genotypes vary across different regions [7]. These disparities are influenced by factors such as geography, population mobility, and sanitation conditions [8].

Molecular epidemiological research is a critical tool for comprehending the dynamics of virus dissemination, transmission, and evolution [9]. The sequencing of viral RNA offers a more precise understanding of the viral genotype and its genetic variations [10]. Moreover, conducting phylogenetic tree analysis using viral sequence data can yield valuable insights into the virus’s origins, transmission routes, and evolutionary pathways [11]. The 5′ untranslated region (UTR), NS5B region, and C region are recognized as core components of HCV, and variations in their sequences are closely linked to the virus’s biological characteristics and epidemiological attributes [12]. Yang et al [13], found that HCV in Guizhou Province is mainly divided into six subtypes: 1a, 1b, 3a, 3b, 6a, and 6n, with subtype 6a being the dominant strain. In the coastal area of Putian, China, subtype 1b dominates, followed by 2a and 1a [4], while in Yunnan Province, subtype 3b is predominant, followed by 3a and 6n [14]. However, the prevalence of HCV in Shandong Province remains insufficiently studied, leaving a significant research gap in this field.

With the rapid advancements in modern bioinformatics technology, machine learning algorithms have found extensive applications in various biomedical domains [15]. In the context of HCV research, machine learning can expedite the classification of extensive viral sequence data and enhance the ability to predict epidemic trends and migration patterns of the virus [16,17]. For example, the Phylogenetic Maximum Likelihood (PhyML) method calculates branch probabilities based on the general time reversible (GTR)+Gamma model and determines the optimal topological structure, which is used to classify the evolutionary relationships of viral subtypes. The Bayesian Markov chain Monte Carlo (MCMC; Bayesian Evolutionary Analysis Sampling Trees [BEAST]) method reconstructs population history using the skyline model and generates posterior distribution trees (maximum clade credibility [MCC] trees) through MCMC sampling to infer the origins and transmission paths of viral subtypes. The exponential clock model uses sample collection time to calibrate the evolutionary tree and estimates the time to the most recent common ancestor (tMRCA) using Bayesian inference, providing estimates of viral divergence times. Although this study did not use traditional supervised learning models (eg, random forests and SVM), it integrates Bayesian statistical inference and maximum likelihood estimation to solve the evolutionary classification, spatiotemporal transmission inference, and divergence time estimation issues of HCV subtypes. These methods enable dynamic analysis of viral evolutionary history through probabilistic modeling and parameter optimization.

Given its economic significance and large population, Shandong Province holds a pivotal role in China. Therefore, conducting comprehensive research on the prevalence of HCV in the province carries significant public health importance [18]. Understanding the trends in prevalence, distribution of primary genotypes, and patterns of HCV transmission in Shandong Province can provide a scientific foundation for disease prevention and control in the region. Furthermore, it can serve as a valuable reference for comprehending the HCV epidemic in other areas of China and globally [19]. Therefore, the goal of this study is to conduct a molecular epidemiology and systematic geography crossover study of HCV isolates from various cities in Shandong using machine learning algorithms. It integrates viral genomics, bioinformatics modeling, and public health policy analysis, aiming to reveal the evolutionary history, transmission networks, and interactions with socioeconomic factors of HCV within the region. This multidimensional integrated analysis provides a scientific basis for regional infectious disease prevention and control.

## Methods

### Subjects

This study collected a total of 320 plasma samples from patients with hepatitis C. The samples were obtained from outpatient and inpatient settings in various hospitals in Shandong Province between 2013 and 2021. The main hospitals include Xintai People’s Hospital, Tai’an Central Hospital, and Dongping People’s Hospital, among others. A total of 38 hospitals participated in sample collection, with further details available in Table S1 in Multimedia Appendix 1. After centrifugation at 3000 rpm for 10 minutes, the obtained plasma samples are divided into 2 tubes and stored at −80 °C. All patients tested positive for anti-HCV antibodies and HCV RNA, with HCV RNA quantification ranging from 1.62×10^3^ to 7.22×10^7^ copies/mL. Serum samples were collected from healthy individuals who tested negative for anti-HCV and were allocated to the negative control group. Serum samples were collected from individuals in the positive control group who were confirmed to have HCV genotypes through sequencing and HCV RNA quantification of ≥10^5^ copies/mL.

Furthermore, the researchers collected baseline demographic information from all participants and evaluated risk factors for HCV infection using a thorough epidemiological questionnaire. The questionnaire covered aspects such as age at diagnosis, gender, occupation, and place of residence. The diagnostic criteria for HCV infection are established according to the 2022 edition of the Chinese Guidelines for the Prevention and Control of Hepatitis C. The study follows the Helsinki Declaration [13].

### Exclusion Criteria

Patients who tested positive for antibodies against the hepatitis B virus, hepatitis D virus (HDV), and HIV were excluded from this study. In addition, patients with known alcohol abuse, drug issues, pregnancy, and those with Child-Pugh grade B or C liver function were not included in this study.

Patients with high-risk factors, such as a history of blood selling, blood transfusion, blood product usage, hemodialysis, intravenous injections, needlestick injuries, maternal infection, high-risk behaviors, or a history of dental treatments, were also excluded [13].

### HCV RNA Extraction and Polymerase Chain Reaction Amplification

Quantitative Polymerase Chain Reaction (qPCR) was performed using the Artus HCV QS-RGQ kit (Qiagen) and the Quant Studio 5 system (Thermo Fisher Scientific). In brief, following the manufacturer’s protocol, viral RNA was reverse transcribed into complementary DNA (cDNA) using the RT-master mixture. A 4-μL aliquot of cDNA was used as a template in a 50-μL PCR reaction mixture. The PCR conditions included an initial incubation at 95 °C for 15 minutes, followed by 50 amplification cycles: denaturation at 95 °C for 30 seconds, annealing at 50 °C for 1 minute, extension at 72 °C for 30 seconds, and a final extension of 5 minutes at 72 °C, followed by a holding step at 37 °C for 1 minute and storage at 4 °C [20]. The primer sequences for the 5′ UTR, Core region, and NS5B region can be found in Table S2 in Multimedia Appendix 1, while specific primer sets for each region are detailed in Tables S3-S5 in Multimedia Appendix 1.

The 5′ UTR, Core region, and NS5B region are helpful in distinguishing different subtypes of HCV [21]. The 5′ UTR refers to the nontranslated region of the viral genome. This region may contain specific sequence variations that can be used for differentiating genotypes. The Core region is the gene region encoding the viral core protein, and its sequence analysis can reveal differences between genotypes and subtypes. The NS5B region encodes the RNA-dependent RNA polymerase of HCV, which plays a critical role in the virus replication process. Sequence analysis of the NS5B region can aid in identifying different genotypes and subtypes [21].

### Sequencing and Phylogenetic Tree Analysis

After separation and amplification of the products through agarose gel electrophoresis, the amplified products of joyous bands (approximately 311 bp in the 5′ UTR, 392 bp in the NS5B region [22], and approximately 503 bp in the C region; Figure S1 in Multimedia Appendix 1) were sent to Qinko Biotechnology Co, Ltd for sequencing. The sequencing data were used for genotyping analysis with National Center for Biotechnology Information’s Basic Local Alignment Search Tool (NCBI BLAST). The sequences obtained from the different primers were renamed based on the sequence information, integrating the original sequence name, sequencing primers, and subtyping information. For instance, the sequence derived from the PV01 primer in the 5′ UTR was renamed P283uPV01-1b. P283u is the original sequence name, PV01 represents the sequencing primer name, and 1b signifies the subtype information.

Subsequently, all sequences from each region were combined with 10 reference strain sequences and were used for evolutionary analysis. The reference strains of HCV include: HCV genotype 1a (M62321), HCV genotype 1b (D90208), HCV genotype 1c (D14853), HCV genotype 2a (D00944), HCV genotype 2b (D10988), HCV genotype 2c (D50409), HCV genotype 2k (AB031663), HCV genotype 3a (D17763), HCV genotype 3b (D49374), and HCV genotype 3k (D63821).

MUSCLE software (Multiple Sequence Comparison by Log-Expectation; version 5.1.linux64; Robert C Edgar) was used for sequence alignment of the 5′ UTR, NS5B, and C regions, applying default parameters. Conserved sequence positions were extracted using Gblocks software (version 0.91b) for further evolutionary analysis. To construct the phylogenetic tree, we applied the GTR+Gamma model and used PhyML software (Phylogenetic Maximum Likelihood; version PhyML 3.3.20190909) to create the maximum likelihood phylogenetic tree. A bootstrap test with 1000 replicates was conducted to validate the results. The tree diagram was visualized and enhanced using EvolView software [23].

### Bayesian Markov Chain Monte Carlo Evolutionary Analysis

This study is based on classifying the major subtypes (1b and 2a) into 2 datasets, each representing a specific HCV subtype. We constructed a maximum likelihood tree using PhyML and imported it into TempEst (Temporal Signal Estimation; v1.5.3), using sample collection year as a covariate. A linear regression analysis was performed between the root-to-tip distance and sample collection time. We observed a correlation coefficient (R²) >0.2, with a positive slope, indicating the presence of a temporal signal in the data that can be used for molecular clock analysis. Therefore, we used the BEAST software package [24]. In this study, we used the Bayesian MCMC algorithm to investigate the ancestral relationships and migration patterns of various HCV subtypes among patients in Shandong Province.

According to research, exponential models perform better than log-normal and strict molecular clock models in HCV sequence analysis [25]. The population model incorporates the Bayesian skyline coalescent model, which surpasses constant size, exponential growth, logistic growth, and expansive growth models [26]. To analyze different HCV genotypes, we used the exponential clock model in combination with the GTR+Gamma+I substitution model and the Bayesian skyline model. The MCMC chain iterates a total of the number of sequences squared multiplied by 3000 times. A tree is generated at intervals of 25,000 iterations.

Each dataset underwent 2 independent MCMC runs, and the effective sample size for each parameter was evaluated using Tracer v1.7.2 software to confirm convergence between chains. The initial generated tree should be aged by 10%, and an MCC tree should be created using TreeAnnotator v1.10.4 software. The resultant posterior tree could be viewed using FigTree v1.4.4 software. Furthermore, this study used the Tracer v1.7.2 software to reconstruct the Bayesian Skyline Plot (BSP) to trace the growth history of HCV in patients with hepatitis C from Shandong province. It also estimated the evolutionary rate and the time of the most recent common ancestor. The BSP is a general skyline graph derived from the target sequence dataset. The distribution of the general skyline graph is then sampled, and these skyline graphs are combined to obtain the posterior distribution of the effective population size over time. Software packages are exclusively obtained from the official BEAST website [13].

### Ethical Considerations

This study was reviewed and approved by the Ethics Committee of Shandong Center for Disease Control and Prevention (institutional review board approval number: SDJK-2021-93-01). All study procedures involving human participants were performed in accordance with the ethical standards of the institutional and national research committees and with the principles of the Declaration of Helsinki (2013 version).

Written informed consent was obtained from all participants prior to the collection of blood samples and epidemiological data. For patients included through secondary data analysis, informed consent had been obtained during the original data collection. The protocol for secondary analysis was separately reviewed and approved by the same ethics committee, and a waiver of reconsent was granted in accordance with institutional policy and relevant regulations.

All data used in the study were fully deidentified prior to analysis to protect participant privacy and confidentiality. No identifiable personal information was collected, recorded, or stored. Data were managed and stored in compliance with institutional data protection policies, and access was restricted to authorized research personnel only.

Participants did not receive monetary or material compensation for participation. All samples and data were collected during routine clinical visits, and no additional interventions or burdens were imposed. The study did not involve vulnerable populations. No identifiable individual images or information are included in the manuscript or supplementary materials. All figures and data are anonymized, and individual identities cannot be inferred in any way.

## Results

### Demographic Characteristics and Age-Occupation Correlation of HCV Infections in the Shandong Region

**Figure 1.**
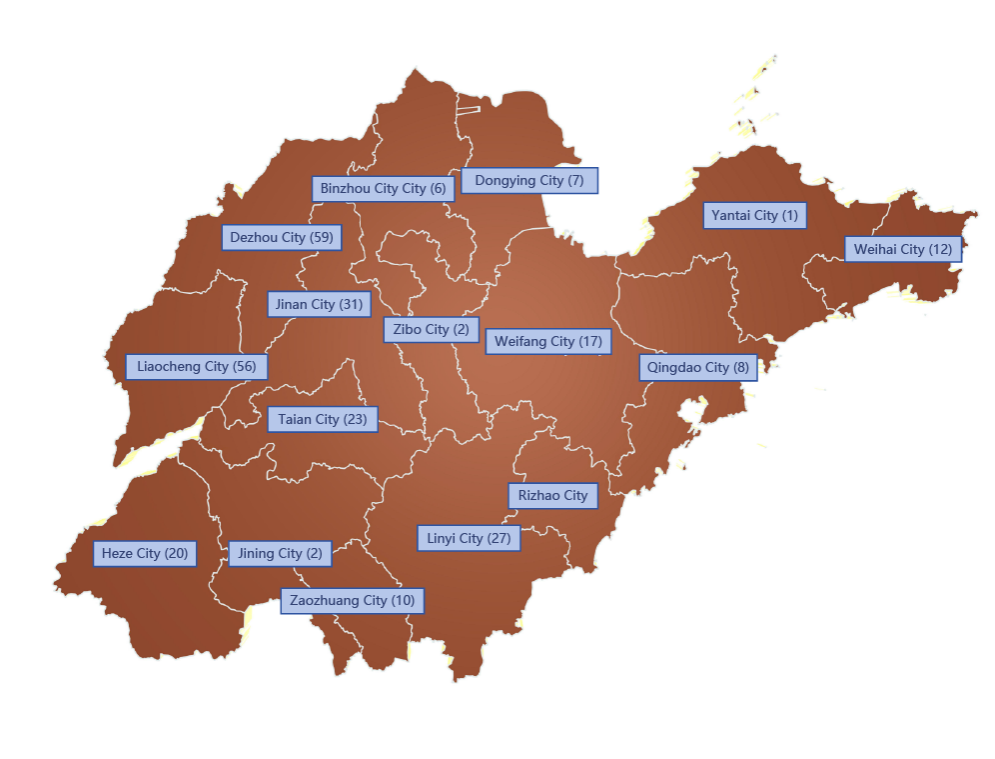
Population distribution of patients with hepatitis C in different regions of Shandong Province.

A total of 320 blood samples from patients infected with HCV in different regions of Shandong, collected from 2013 to 2021, were included based on the established exclusion criteria. Excluding the few patients with incomplete identity information, the population’s demographic characteristics are displayed in Figure 1 and Table 1. The data reveal 161 (50.3%) males and 121 (37.8%) females in the sample. The patients included in this study were HCV positive; their ages ranged from 7 to 80 years, with a mean age of 54.3 (14.3) years. The prevalence of HCV infection is approximately 7.8% in the age group under 30 years, 23.8% in the age group between 30 and 49 years, 54.9% in the age group between 50 and 69 years, and 13.5% in the age group 70 years and older. Analysis of the age-specific data clearly shows a higher frequency of HCV infection in the 50‐69 years age group. Furthermore, when excluding patients who have not yet received a clinical diagnosis, it is observed that 97.6% of patients infected with HCV suffer from chronic hepatitis. This condition is particularly prevalent among farmers (75.2%), with homemakers and unemployed individuals at home exhibiting the next highest prevalence rates.

**Table 1.** Demographic characteristics of hepatitis C virus-positive patients, 2013-2021, Shandong, China.

Characteristics	Total patients (N=320), n (%)
Sex	
Male	161 (50.31)
Female	121 (37.81)
Unknown	38 (11.88)
Age (years)	
<30	22 (7.8)
30‐39	23 (8.2)
40‐49	44 (15.6)
50‐59	86 (30.5)
60‐69	69 (24.4)
≥70	38 (13.5)
Unknown	38 (11.88)
Disease type	
Chronic	245 (97.6)
Acute	6 (2.4)
Unknown	69 (21.56)
Occupation	
Farmer	212 (75.2)
Nonfarmers	70 (24.8)
Unknown	38 (11.88)

### Methodology and Success Rates of HCV Genotyping in the 5′ UTR, Core Region, and NS5B Region With Sequence Variability Analysis

The genome of HCV comprises a 5′ UTR, which contains a highly conserved RNA structure. This region also includes a single open reading frame responsible for encoding structural proteins (Core, E1, and E2) and nonstructural proteins (p7, NS2, NS3, NS4A, NS4B, NS5A, and NS5B). A 3′ UTR is also present within the genome [27]. HCV’s 5′ UTR is highly conserved, exhibiting minimal sequence variability. Hence, it is well-suited for the development of specific primers for amplification purposes. This region exhibits a high detection rate and is crucial in HCV RNA quantification and genotyping. However, several studies have identified limitations in using the 5′ UTR for distinguishing HCV genotypes, as the misclassification of 1a as 1b is quite prevalent [28]. Types 1, 2, 3, and 4 cannot be fully differentiated solely by their subtypes [29]. Certain 6-type sequences in the Southeast Asian region closely resemble the 1a and 1b sequences. As a result, it is not uncommon for 6-type sequences to be mistakenly classified as 1a and 1b [17]. In the species-specific region of the HCV genome, such as the C, E1, and NS5B regions, there is a high degree of heterogeneity between genotypes and subtypes, with gene sequences containing valuable information for genotyping. Therefore, sequence analysis based on this region can accurately differentiate HCV subtypes and even different isolates within the same subtype. Given the situation above, scholars have proposed a combined approach that uses 5′ UTR genotyping methods along with genotyping of the Core or NS5B region in recent years. This approach improves the detection rate and accurately distinguishes between HCV genotypes [30].

We primarily focused on sequencing the 5′ UTR. In addition, we used sequencing of the NS5B and Core regions as supplementary methods for genotyping analysis of 320 patients. A total of 320 gene fragments from the 5′ UTR were successfully amplified and sequenced, yielding a success rate of 98.8% (320/324). Similarly, 282 gene fragments from the Core region were successfully amplified and sequenced, resulting in a success rate of 89.5% (282/315). Furthermore, 233 gene fragments from the NS5B region were successfully amplified and sequenced, with a success rate of 77.7% (233/300). Consistent with previous studies, the amplification frequencies vary among the three regions, with the highest frequency observed in the 5′ UTR, followed by the Core region, and the lowest frequency observed in the NS5B region. A total of 104 cases of amplification failure may be attributed to low viral load and weak primer specificity.

**Figure 2.**
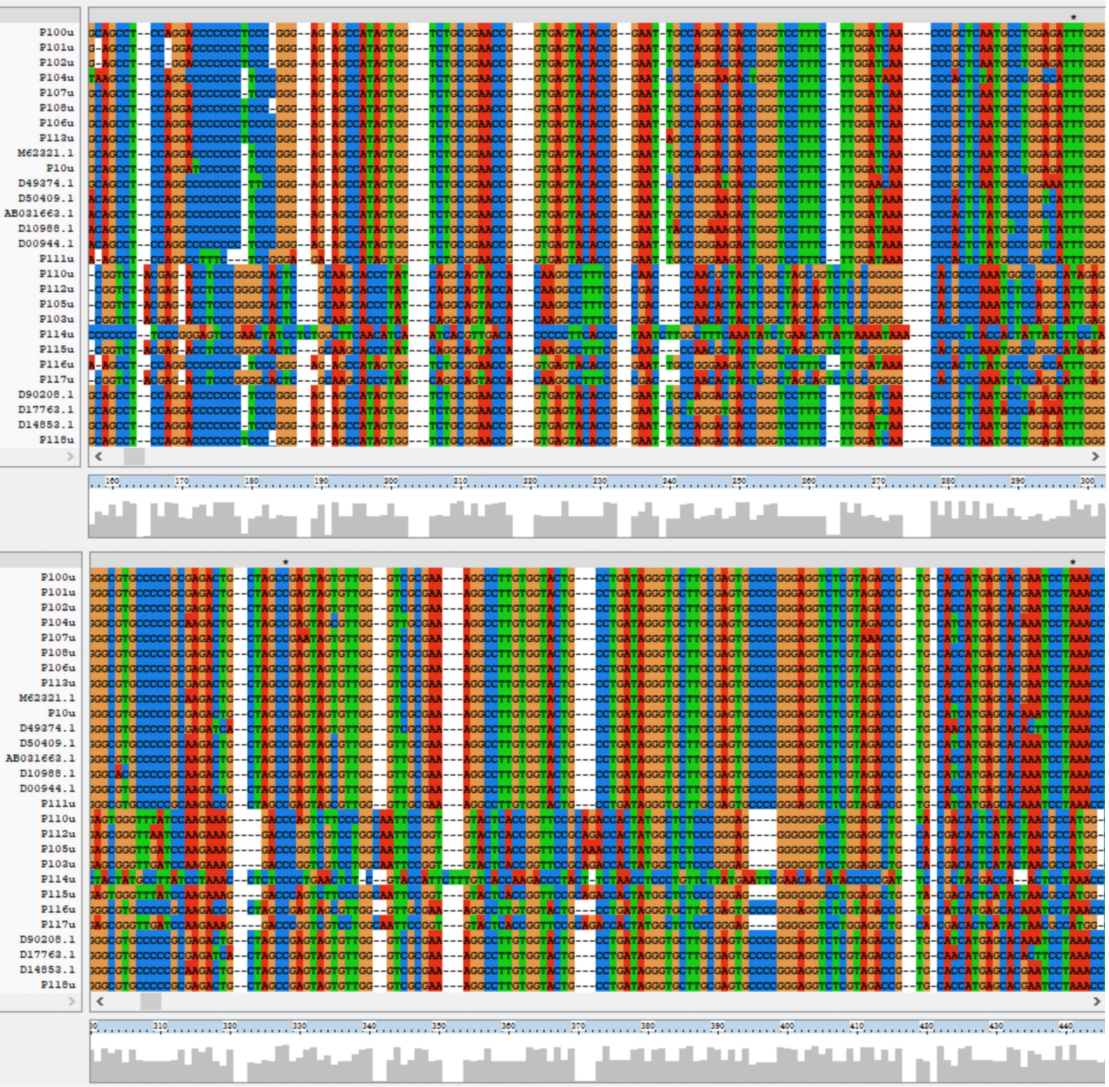
Comparison of 307 cases’ 5' untranslated region nucleotide sequences with reference sequences in the GenBank database.

After successful amplification, the resulting sequences were used as input for NCBI BLAST to search for the most likely similar sequences, thus determining the genotype of each region. Taking the P4u sequence from the 5′ UTR as an example, the BLAST analysis results (Table S6 in Multimedia Appendix 1) showed that the nucleotide homology (PNI) of the P4u sequence was 100%, with the highest score of 414. This 280 bp fragment showed 100% similarity to the HCV isolate QC156 (Accession: KJ439771.1) from nt32 to nt311, 100% similarity to the HCV subtype 1b strain MD29 (Accession: AF207770.1) from nt85 to nt364, and 99.29% similarity to the HCV open reading frame gene (Accession: D90208.1) from nt85 to nt364. A multiple sequence alignment was performed on 307 partial gene sequences from the 5′ UTR using ClustalX2 software and compared to 10 reference strain sequences from the GenBank database. The nucleotide sequences of the 5′ UTR are considered highly conserved; however, as shown in Figure 2, there were some mutations and variations in the 5′ UTR of different HCV genotypes.

### Distribution of HCV Genotypes, Potential Recombinants, and Correlation With Gender and Age

A total of 307 cases were genotyped for the 5′ UTR of the gene, 280 cases for the Core region, and 233 cases for the NS5B region. The typing results are presented in Table 2. It is evident that the 5′ UTR exhibits a high amplification frequency; however, distinguishing between many gene segments is challenging in the typing comparison. Conversely, while the amplification frequencies of the Core region and NS5B region are relatively low, they demonstrate superior capability in analyzing gene subtypes. The detection sensitivity was improved after conducting a combined analysis of the three. Four samples that did not amplify in the 5′ UTR could differentiate genotypes through successful amplification in the Core and NS5B regions. Moreover, gene fragments with ambiguous genotyping in the 5′ UTR could be further classified into subtypes.

**Table 2.** Results of Hepatitis C virus segmentalization and combined analysis in patients with hepatitis C.

Genotype	HCV^a^ genotype											
	Genotype 1^b^				Genotype 2^b^			Genotype 33b	Genotype 6^b^			Total
	1a	1b	1a/1b	1b/2k,2a	2*^c^	2a	2a,2k/1b		6a	6k	6u	
5’ UTR^d^	2	170	5	1	50	75	1	1	1		0	307
Core	1	160	0	0	0	117	0	0	1	1	1	280
NS5B	1	134	0	0	0	96	0	0	1		1	233
Joint analysis	312											

a HCV: Hepatitis C virus.

b Types 1, 2, and 6 all contain different numbers of recombinant cases, totaling 13 cases.

c *The number of cases indicates that this type cannot further differentiate subtypes.

d 5′ UTR: 5′ untranslated region.

**Figure 3.**
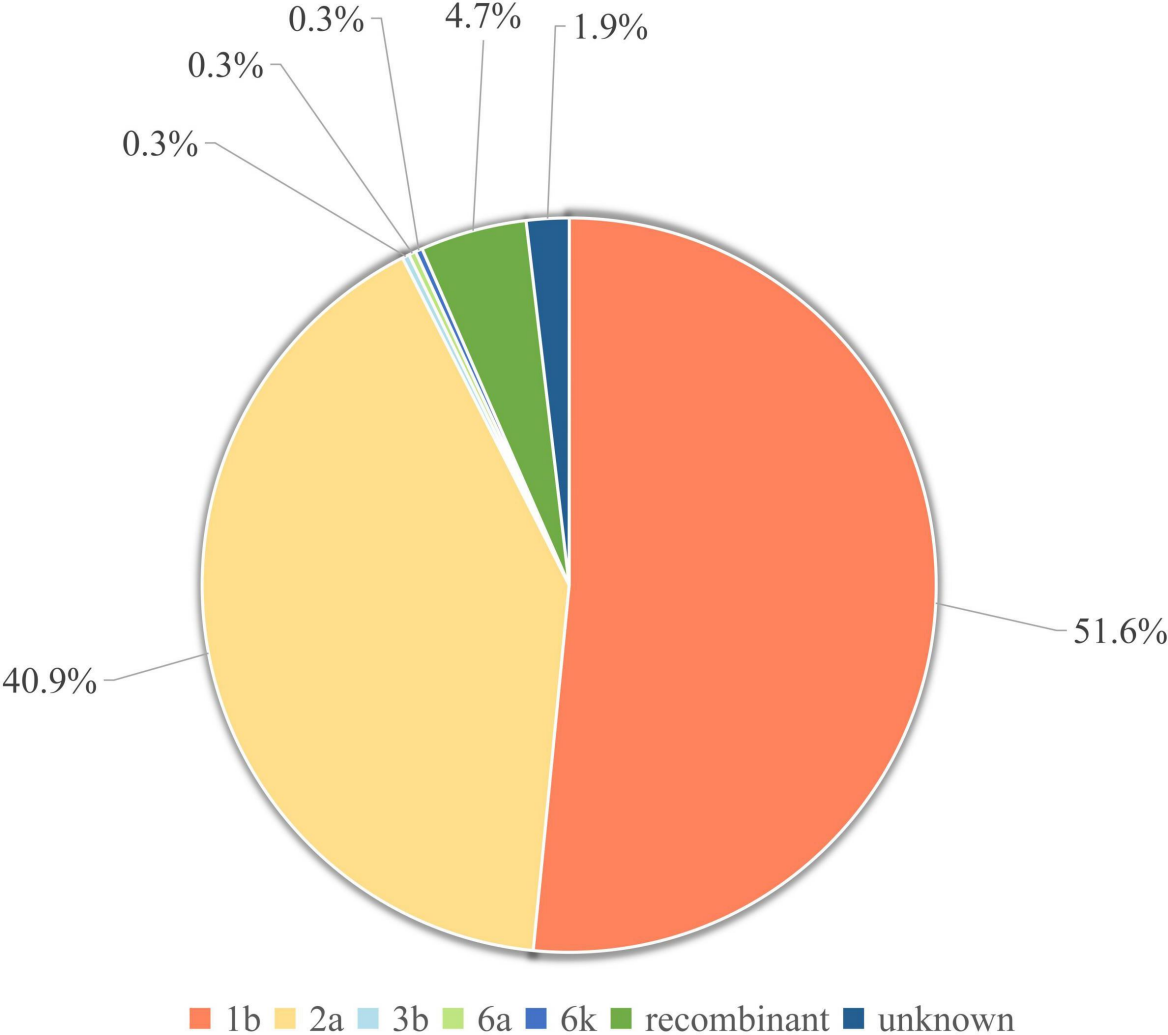
Distribution of HCV genotypes in 320 hepatitis C patients with hepatitis C.

The main genotypes of HCV prevalent in most regions of our country are genotype 1b, followed by genotype 2a. These results are confirmed by our typing analysis (Figure 3). Among the 320 patients with hepatitis C analyzed, the most common HCV subtypes include 1b, 2a, 3b, 6a, and 6k. Of these subtypes, 1b (n=165) is the most dominant, followed by 2a (n=131), 3b (n=1), 6a (n=1), and 6k (n=1). Since 2002, Kalinina et al [31] reported the first case of the HCV natural recombinant 2k/1b in 2002, and several researchers have identified different recombinant strains through multiregion genotyping. This study found 13 cases with inconsistent genotyping in the 5′ UTR, Core region, and NS5B region, which are preliminarily considered potential recombinant strains of HCV, pending further confirmation in subsequent studies. In addition, there are 8 cases whose genotypes remain unclear.

As demonstrated in Table 3, with the exclusion of a few patients with incomplete identity information, the HCV genotype ratio is higher in females compared to males. Out of the 148 patients diagnosed with genotype 1b, the average age was 54.7 (14.0) years. Among them, 86 were males (58.1%) and 62 were females (41.0%). One hundred eleven patients with genotype 2a were included in the study, with an average age of 54.9 (14.4) years. The patient population comprised 57 males (51.4%) and 54 females (48.6%). Based on statistical analysis, there was no difference in the average age of patients infected with genotypes 1b and 2a of HCV (*P*>.05).

**Table 3.** Correlation between clinicopathological features and hepatitis C virus genotype frequencies among 282 patients with hepatitis C.

Clinicopathological feature	HCV^a^ genotype					
	Genotype 11b	Genotype 22a	Genotype 33b	Genotype 6		Recombinant
				6a	6k	
Sex, mean (SD)						
Male	86 (58.1)	57 (51.4)	0	0	1	3
Female	62 (41.0)	54 (48.6)	1	1	0	9
Age (years), mean (SD)						0
<30	10 (6.8)	7 (6.3)	0	0	0	1
30‐39	10 (6.8)	13 (11.7)	0	0	0	0
40‐49	24 (16.1)	14 (12.6)	1	1	0	2
50‐59	46 (31.1)	34 (30.6)	0	0	1	4
60‐69	38 (25.7)	26 (23.4)	0	0	0	4
≥70	20 (13.5)	17 (15.3)	0	0	0	1
Age (years), mean (SD)	54.7 (14.0)	54.9 (14.4)				55.3 (12.8)
Total, mean (SD)	148 (54.0)	111 (40.5)	1 (0.4)	1 (0.4)	1 (0.4)	12 (5.8)

a HCV: hepatitis C virus.

The youngest patient diagnosed with hepatitis C is a 7-year-old girl with HCV genotype 1b, while the oldest patient is an 87-year-old woman with HCV genotype 1b. No deviation was observed in the age-specific incidence rates of the main HCV genotypes (1b, 2a) in this study. Most patients with hepatitis C are older than 50 years, with genotype 1b accounting for 70.3% and genotype 2a accounting for 69.3%. There is a lower prevalence in patients younger than 50 years, with 29.7% having genotype 1b and 30.7% having genotype 2a.

### Phylogenetic Analysis of HCV Sequences Using Maximum Likelihood and Genotype Clustering Validation

**Figure 4.**
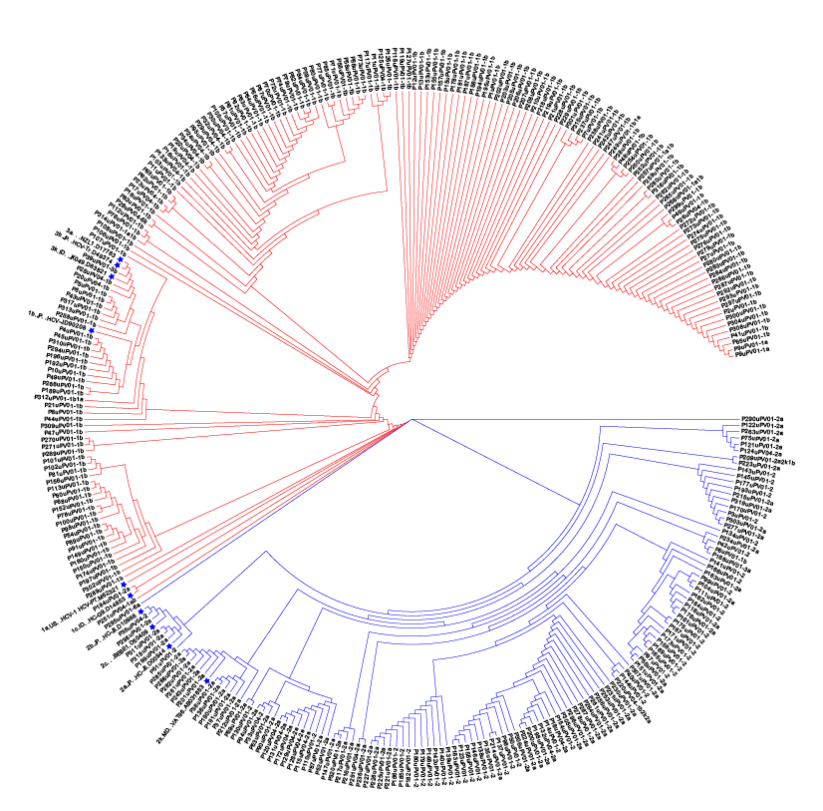
Evolutionary tree analysis of nucleotide sequences in the 5′ untranslated region. Sequences marked with asterisks represent reference strain sequences; branch lengths are proportional to the evolutionary distance and scale between sequences.

We merged the 10 reference strain sequences obtained from the GenBank database with 307 5′ UTR sequences, 280 Core region sequences, and 233 NS5B region sequences. We selected the GTR+Gamma model and used the PhyML software to construct a maximum likelihood phylogenetic tree based on these sequences. We repeated the sequence alignment 1000 times using Bootstrap. The phylogenetic analysis of the 5′ UTR, Core region, and NS5B region circular system revealed substantial diversity among HCV isolates belonging to different genotypes, as demonstrated by Figure 4 and Figure S2-S3 in Multimedia Appendix 1. Furthermore, the clustering of genotypes 1b and 2a provided additional validation for the accuracy of the NCBI Blast results.

### Geographical Dissemination, Estimation of the Most Recent Common Ancestor, and Population Dynamics of HCV Subtypes 1b and 2a in Shandong Province

To estimate the evolutionary relationship and emergence time of the 2 major HCV genotypes (1b and 2a) among patients with hepatitis C in different cities of Shandong Province, we conducted an evolutionary analysis using the highly conserved 5′ UTR sequences, taking into account their conservation in the region.

**Figure 5.**
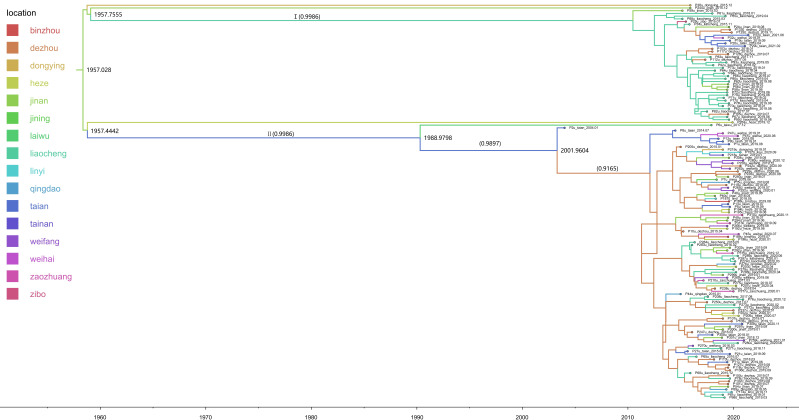
Maximum clade credibility tree based on the 5' untranslated region of subtype 1b. The time scale on the x-axis represents years (1960‐2020). The branches of the tree are colored based on the sampling city in Shandong Province (as shown in the legend). Only nodes with Bayesian posterior probabilities > 0.9 are shown, with posterior support values in parentheses. The phylogenetic tree can be roughly divided into two major clusters (Cluster I and Cluster II), each showing high geographic clustering and clear transmission paths. In Cluster I, the viral strains from the Liaocheng region show strong clustering, with a relatively limited transmission range. In Cluster II, viral strains from Tai’an and Dezhou are radially distributed, potentially playing a “bridge” role in the transmission of the 1b subtype. There is a noticeable temporal structure between the viral strains from multiple cities, reflecting the long-term dynamics of HCV transmission.

A systematic geographical analysis of the 1b subtype revealed a pronounced genetic relationship among 1b isolates from various cities in Shandong Province. Jinan may be the origin of the 1b subtype in Shandong, dating its divergence back to 1957 (Figure 5). The MCC tree of sequence 1b is roughly divided into 2 clusters, exhibiting a high posterior probability. The 2a subtypes of Cluster I primarily spread in the Licheng region, with a few cases extending to other urban areas. Regarding the transmission of the 2a subtypes in Cluster II, Taian and Dezhou have acted as conduits, enabling the successive spread of the 2a subtypes to surrounding cities.

**Figure 6.**
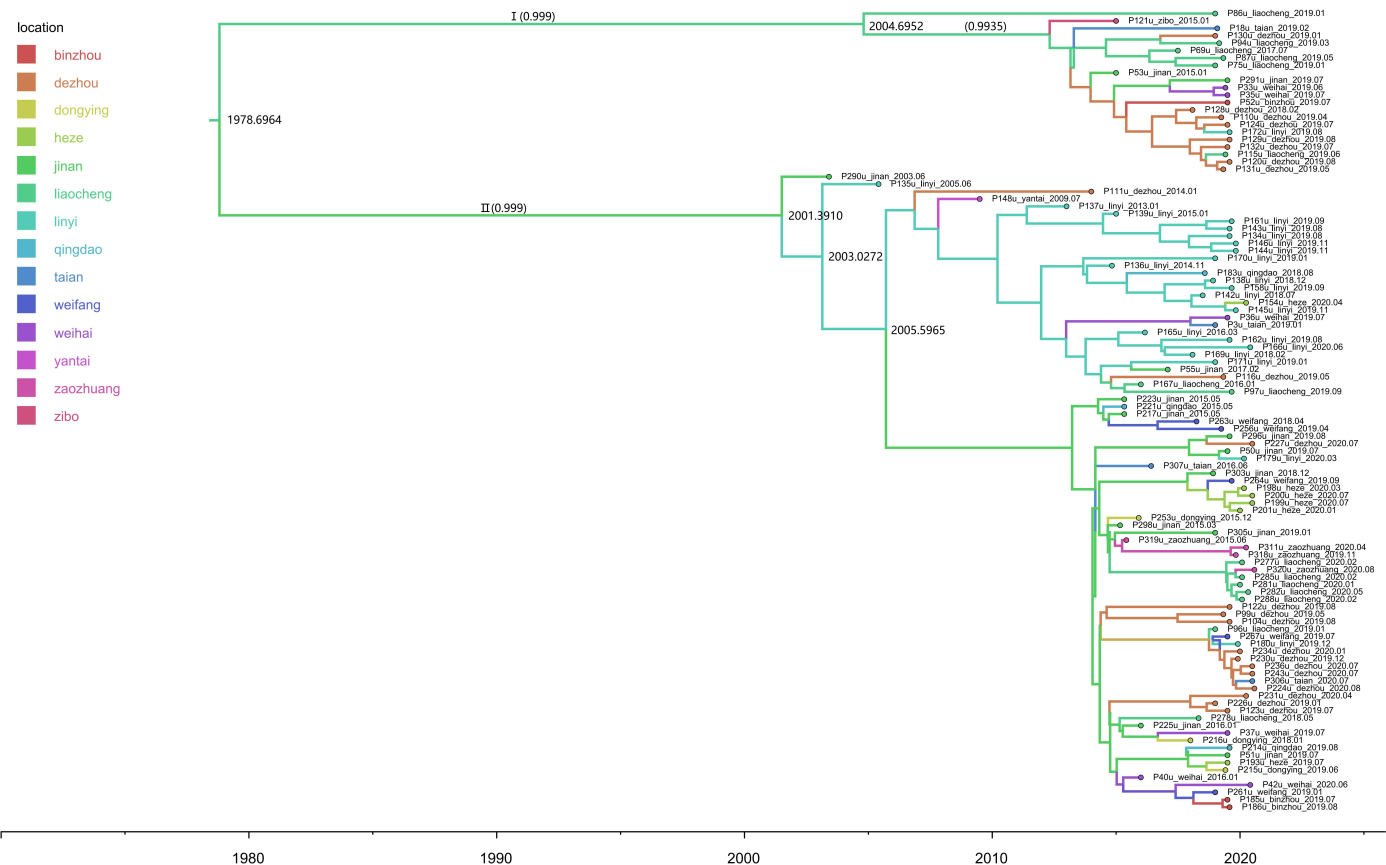
Maximum clade credibility tree based on the 5' untranslated region of subtype 2a. The time scale on the x-axis represents years (1980‐2020), and the branches of the tree are colored based on the sampling cities in Shandong Province (as indicated in the legend). Only nodes with Bayesian posterior probabilities >0.9 are displayed, with posterior support values in parentheses. The tree structure can be roughly divided into two major clusters (Cluster I and Cluster II), each showing high confidence (posterior probability of 0.999). This suggests that the HCV subtypes in Shandong may have undergone at least 2 major diffusion events: Cluster I: Centered around the Liaocheng region, showing a certain degree of local transmission clustering. Cluster II: Exhibiting a multi-region mixed distribution, with Tai’an and Dezhou positioned as key nodes in the transmission network, potentially acting as “bridges” in the spread of the 2a subtype.

In the phylogenetic analysis of subgenotype 2a (Figure 6), we divided the MCC tree of 2a sequences into two clusters, both exhibiting a high posterior probability. Both clusters suggest that Jinan and Liaocheng could have been bridge points in transmitting the 2a variant. The dissemination of the 2a variant to neighboring cities could be traced back to 1979.

**Figure 7.**
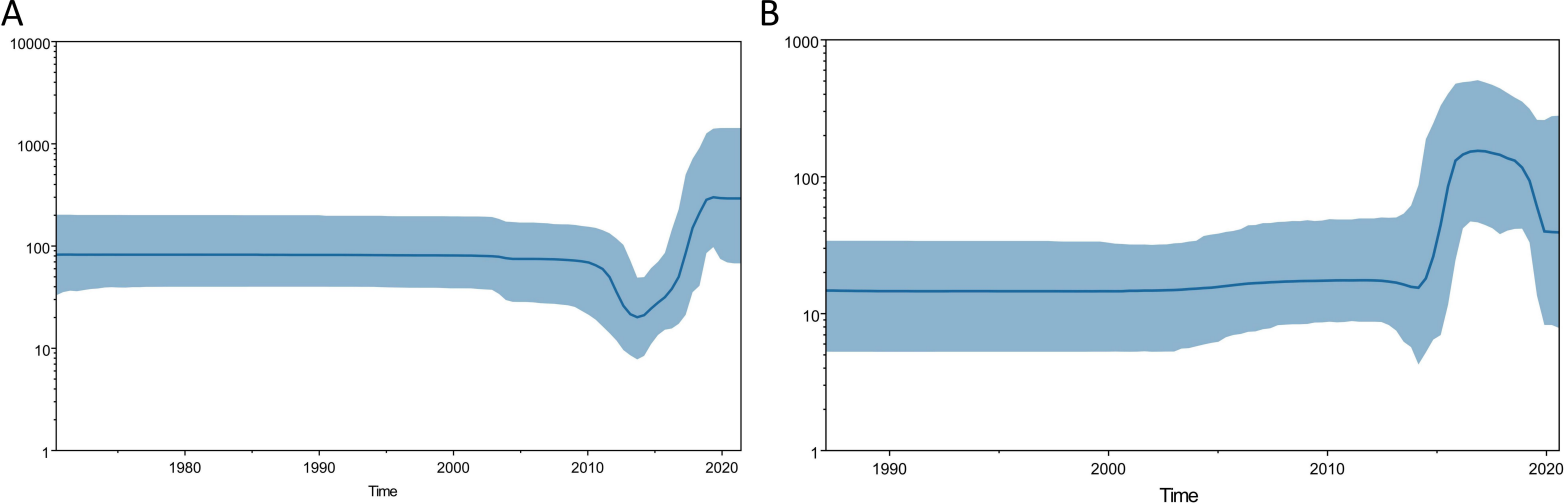
Bayesian skyline plot of the 2 major hepatitis C virus subtypes in Shandong. Graphs A and B represent HCV genotypes 1b and 2a, respectively. The horizontal ruler (x-axis) at the bottom displays years, while the vertical ruler (y-axis) on the left displays effective population size on a logarithmic scale. The black curve represents the median estimate of the changing effective population size over time. The blue curve with upper and lower areas defines the 95% highest posterior density confidence interval.

Subsequently, we performed a Bayesian skyline analysis to estimate the most recent common ancestor (tMRCA) time and the growth dynamics for these 2 prominent HCV subtypes (Figure 7). tMRCA for subtype 1b is estimated to be 1957 (95% CI 1943‐1973), and for subtype 2a, it is estimated to be 1979 (95% CI 1967‐1989). We used the Tracer software to generate a BSP to investigate the rapid expansion stage in the viral population. The rapid growth of phases 1b and 2a occurred from 2014 to 2019 and from 2014 to 2016, respectively. Before these rapid phases, the population sizes of 1b and 2a had remained relatively stable for the past 40 and 30 years. Furthermore, both population groups 1b and 2a underwent a decrease in size. The rapid decline stage of 1b (2010‐2014) preceded the rapid growth stage, whereas the rapid decline stage of 2a (2018‐2020) followed the rapid growth stage. Until now, there has been a relatively stable trend in the population sizes of 1b and 2a.

## Discussion

### Principal Findings

This study uses advanced phylogenetic and machine learning algorithms to perform genotype classification and evolutionary analysis of HCV isolates [32]. Previous research primarily relied on traditional molecular biology techniques, including nested PCR and gene sequencing [33]. By integrating machine learning algorithms, we enhance analysis accuracy, processing speed, and efficiency when dealing with complex data [34]. This technological innovation facilitates more precise migration and evolution tracking, providing robust data support for future disease control strategies [35].

We collected serum samples from 320 patients with hepatitis C in Shandong Province and successfully sequenced the 5 UTR from all 320 cases, the Core region from 282 cases, and the NS5B region from 233 cases. The predominant HCV subtypes in Shandong were 1b and 2a. The early spread of HCV 1b and 2a in Shandong occurred primarily in Jinan and Liaocheng, with population trends exhibiting stability, growth, and decline over time. Although the 1b subtype is globally widespread, other subtypes may be more common in regions such as Europe and North America [36]. This finding suggests that different geographical locations may have unique viral epidemic patterns, which is crucial for developing localized disease prevention strategies.

### Comparison to Previous Work

While subtype 1b is widely distributed worldwide, certain regions, such as Europe and North America, may exhibit a higher prevalence of other subtypes [36]. This discovery underscores the significance of recognizing distinct geographical patterns in viral outbreaks, which is essential for developing localized disease prevention and control strategies. Using Bayesian phylogenetic inference, we estimated the tMRCA (time to the most recent common ancestor) for the 1b and 2a subtypes in Shandong Province to be 1957 and 1979, respectively. The spread of HCV 1b and 2a subtypes closely correlates with the socioeconomic development of Shandong. The origin of the 1b subtype in 1957 likely corresponds with the first systemic public health interventions post-1949 in China, during the “golden age” of public health system construction. During the late 1950s, China implemented large-scale vaccination, patriotic health campaigns, and grassroots health care network development, which increased the frequency of contact between medical institutions and populations, potentially elevating the risk of bloodborne pathogen transmission [37]. Conversely, the 1979 spread of the 2a subtype reflects the consequences of medical resource imbalance during China’s market-oriented reforms. The rapid urbanization and frequent population movement in Shandong, coupled with the concentration of medical resources in cities and limited rural health care facilities, may have increased the occurrence of high-risk behaviors, such as paid blood donations and unsafe injections, contributing to the spread of the virus [37]. Both Jinan and Liaocheng played pivotal roles in these diffusion events, highlighting the central role of major cities in pathogen transmission.

### Limitations

First, the time span is limited, while the study covers samples collected from 2013 to 2021, this timeframe may not be sufficient to fully capture the long-term evolution of a chronic viral disease like HCV, which has a prolonged latency period and complex evolutionary history. Certain subtype introductions may have occurred earlier than the period observed, which could introduce uncertainty in the tMRCA estimation.

Second, there is a potential bias in the sample source: all samples were collected from patients attending medical institutions, which may introduce biases as individuals with mild symptoms, asymptomatic infections, or high-risk but undiagnosed individuals were likely excluded, potentially limiting the representativeness of the sample in terms of age, sex, and transmission route.

Third, there are limitations in the technical methods: although this study uses the BEAST Bayesian MCMC method and Bayesian skyline plots, these methods rely on certain assumptions (such as molecular clock assumptions, substitution model settings, and sample representativeness) and may introduce biases when facing uneven sequence distributions, weak time signals, or recombination events. In addition, machine learning and maximum likelihood methods for genotyping and phylogenetic tree construction may be limited by input data quality.

Fourth, the identification of recombinant strains remains uncertain: some samples identified as potential recombinants have not been validated by whole-genome sequencing or recombination detection algorithms, so the current conclusions should be interpreted cautiously.

### Future Directions

Future research should aim to address several limitations identified in this study. First, expanding the temporal scope to include earlier samples would improve estimates of long-term viral evolution and help clarify the historical introduction of HCV subtypes in the region. In addition, community-based sampling—including asymptomatic individuals and high-risk populations not captured in hospital-based studies—would enhance the representativeness and generalizability of findings.

Incorporating whole-genome sequencing and advanced recombination detection tools would also enable more accurate identification of recombinant strains, particularly those with inconsistent genotyping results across genomic regions. Applying and comparing a broader range of machine learning algorithms may further enhance the efficiency and precision of genotypic classification and transmission modeling.

Moreover, future studies could explore the application of these approaches at a national scale by including data from other provinces in China. Such expansion would provide a more comprehensive understanding of HCV’s genetic and geographic diversity across the country. Finally, interdisciplinary collaboration among experts in virology, public health, clinical medicine, and bioinformatics will be essential to translate molecular epidemiological insights into targeted intervention strategies, such as genotype-specific treatments and vaccine development.

### Conclusions

This study investigated the genetic diversity and dissemination patterns of HCV in Shandong Province through sequencing and phylogenetic analysis. Genotypes 1b and 2a were identified as the predominant subtypes, with evidence of localized transmission and multiple diffusion events. Temporal and geographic reconstructions suggest distinct expansion phases for these subtypes, particularly between 2014 and 2019. The findings offer insights into regional HCV epidemiology that may support public health strategies in Shandong and similar settings. However, interpretations should be made in light of the study’s methodological and temporal limitations.

## Supplementary material

10.2196/60207 Multimedia Appendix 1 Additional figures and tables.

## Abbreviations list

BEAST: Bayesian Evolutionary Analysis Sampling Trees
BSP: Bayesian skyline plot
GTR: general time reversible
HCV: hepatitis C virus
HDV: hepatitis D virus
MCC: maximum clade credibility
MCMC: Markov chain Monte Carlo
MUSCLE: Multiple Sequence Comparison by Log-Expectation
NCBI BLAST: National Center for Biotechnology Information Basic Local Alignment Search Tool
PhyML: Phylogenetic Maximum Likelihood
qPCR: quantitative polymerase chain reaction
tMRCA: time to the most recent common ancestor
UTR: untranslated region
