# Supplementary material for: Genotype Distribution and Migration Patterns of Hepatitis C Virus in Shandong Province, China: Molecular Epidemiology and Phylogenetic Study
Source: JMIR Med Inform. 2025 Aug 18;13:e60207. doi: 10.2196/60207 (PMC12360734; doi:10.2196/60207)
Supplement: Multimedia Appendix 1 [file medinform-v13-e60207-s001.docx]

**Table S1. List of Hospitals Participating in Sample Collection**

| Hospital Name | Count (n = 320) | Percentage (%) |
| --- | --- | --- |
| Binzhou Binhai District People's Hospital | 5 | 1.6% |
| Caoxian People's Hospital | 4 | 1.3% |
| Chengwu People's Hospital | 4 | 1.3% |
| Dezhou Lingcheng District People's Hospital | 6 | 1.9% |
| Dingtao District People's Hospital | 9 | 2.8% |
| Dongming People's Hospital | 2 | 0.6% |
| Dongping People's Hospital | 4 | 1.3% |
| Dongying Dongying District People's Hospital | 2 | 0.6% |
| Gaotang People's Hospital | 2 | 0.6% |
| Huimin People's Hospital | 4 | 1.3% |
| Leiling People's Hospital | 10 | 3.1% |
| Liaocheng Dongchangfu People's Hospital | 16 | 5.0% |
| Linqing People's Hospital | 2 | 0.6% |
| Linyi People's Hospital | 49 | 15.3% |
| Linyi County People's Hospital | 6 | 1.9% |
| Ningjin People's Hospital | 6 | 1.9% |
| Pingyuan People's Hospital | 8 | 2.5% |
| Qihe People's Hospital | 8 | 2.5% |
| Qingdao Huangdao District Central Hospital | 4 | 1.3% |
| Qingdao City Hospital | 4 | 1.3% |
| Rongcheng People's Hospital | 6 | 1.9% |
| Rushan People's Hospital | 4 | 1.3% |
| Shandong University Qilu Hospital Dezhou | 8 | 2.5% |
| Shandong Provincial Public Health Clinical Center Baoshan District | 27 | 8.4% |
| Shandong Qianfoshan Hospital | 2 | 0.6% |
| Shenxian People's Hospital | 34 | 10.6% |
| Tai'an Central Hospital | 15 | 4.7% |
| Weihai City Hospital | 13 | 4.1% |
| Wucheng People's Hospital | 4 | 1.3% |
| Xiaojin County People's Hospital | 2 | 0.6% |
| Xintai People's Hospital | 15 | 4.7% |
| Yantai Qishan Hospital - Yantai Infectious Disease Hospital | 2 | 0.6% |
| Yanggu County People's Hospital | 17 | 5.3% |
| Yucheng People's Hospital | 8 | 2.5% |
| Yuncheng People's Hospital | 2 | 0.6% |
| Zibo Infectious Disease Hospital | 2 | 0.6% |
| Zoucheng People's Hospital | 2 | 0.6% |
| Zouping People's Hospital | 2 | 0.6% |

**Table S2. 5'-UTR, NS5B and C region PCR amplification primers**

| **Amplification segment** | **Name of primer** | **Primer sequence (5′-3′)** |
| --- | --- | --- |
| 5’UTR  Forward primer  Reverse primer | PV01  PV04 | 5′-CGCAGAAAGCGTCTAGCCAT-3′  5′-TTTGGTTTTTCTTTGAGGTTTAGGA-3′ |
| NS5B  Forward primer  Reverse primer | CX-NS51  CX-NS52  CX-NS53  CX-NS54  CX-NS59 | 5′-TATGACACCCGCTGYTTYGACTC-3′  5′-TATGAYACCCGMTGCTTTGACTC-3′  5′-CCGATGGGTTTCTCCTATGAYAC-3′  5′-CCCATGGGCTTYTCYTAYGACAC-3′  5′-GGTCATAGCCTCCGTGAADRCTC-3′ |
| C region  Forward primer  Reverse primer | CX-C51  CX-C3 | 5′-TCCTAAACCTCAAAGAAAAACCAAA-3′  5′-AACCRGGNARRTTCCCTGTTGC-3′ |

**Table S3. 5 '-UTR region sequencing system**

| **Reagent name** | **Reagent dosage** |
| --- | --- |
| 5×RT-PCR Buffer | 10μL |
| Enzyme Mix | 2μL |
| PV01 (100μM) | 2μL |
| PV04 (100μM) | 2μL |
| Template (RNA) | 10μL |
| Water | 24μL |
| Total | 50μL |

**Table S4. NS5B region sequencing system**

| **Reagent name** | **Reagent dosage** |
| --- | --- |
| 5×RT-PCR Buffer | 10μL |
| Enzyme Mix | 2μL |
| CX-NS51 (100μM) | 2μL |
| CX-NS52 (100μM) | 1μL |
| CX-NS53 (100μM) | 1μL |
| CX-NS54 (100μM) | 1μL |
| CX-NS59 (100μM) | 1μL |
| Template (RNA) | 10μL |
| Water | 22μL |
| Total | 50μL |

**Table S5. Core region sequencing system**

| **Reagent name** | **Reagent dosage** |
| --- | --- |
| 5×RT-PCR Buffer | 10μL |
| Enzyme Mix | 2μL |
| CX-C3 (100μM) | 2μL |
| CX-C51 (100μM) | 2μL |
| Template (RNA) | 10μL |
| Water | 24μL |
| Total | 50μL |

**Table S6 . NCBI nucleotide BLAST results for the sequence in the 5’UTR region of HCV P4u sequence (partial alignment of the results)**

| **Description** | **Max score** | **E-value** | **Identity** | **Accession** |
| --- | --- | --- | --- | --- |
| Hepatitis C virus isolate QC156  Hepatitis C virus subtype 1b strain MD29  Hepatitis C virus subtype 1b strain MD11  Hepatitis C virus subtype 1b genomic RNA  Hepatitis C virus ORF gene | 518  518  518  512  507 | 3e-142  3e-142  3e-142  2e-140  6e-147 | 100%  100%  100%  99.64%  99.29% | KJ439771.1  AF207770.1  AF207752.1  LC011930.1  D90208.1 |


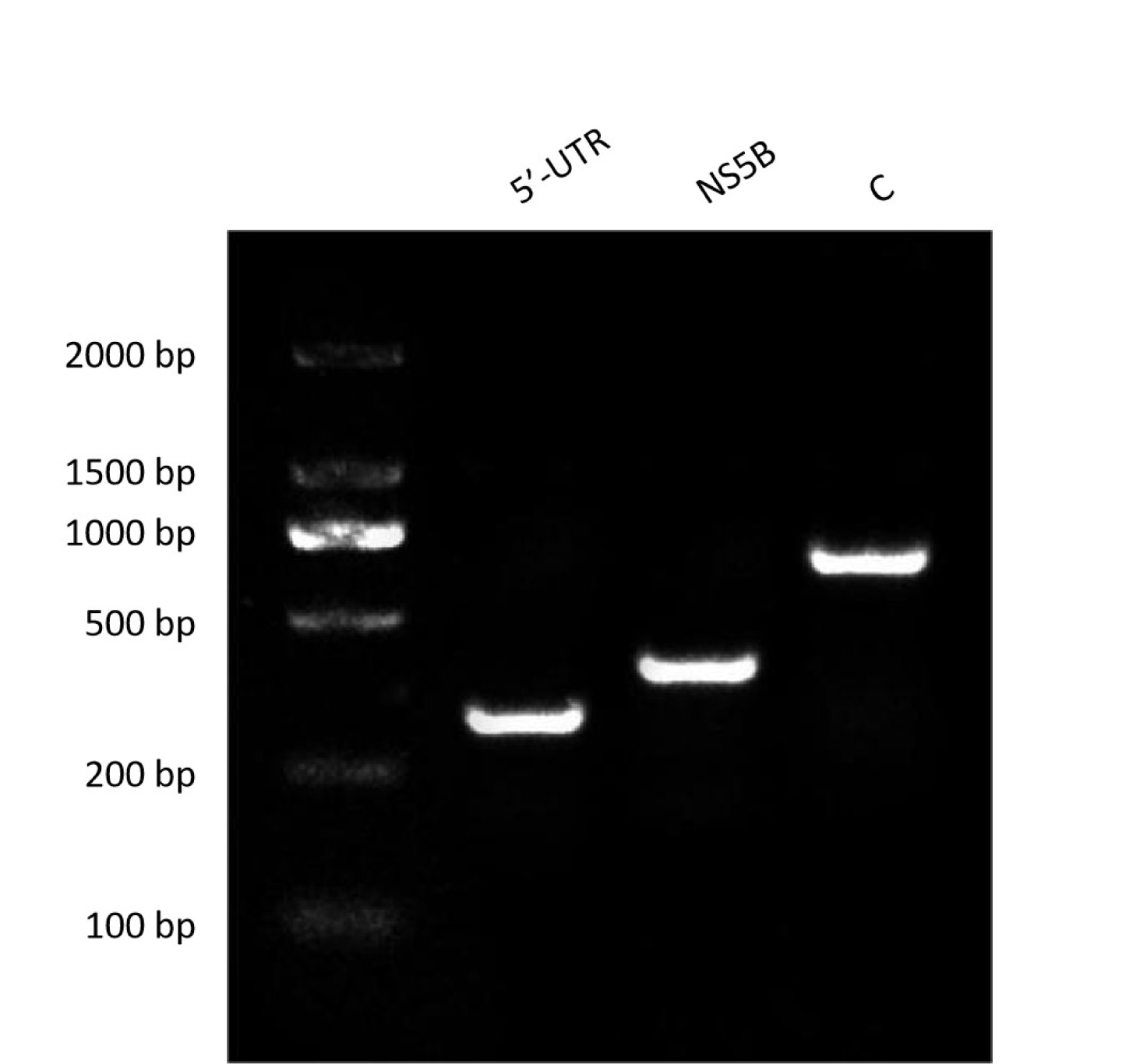


**Figure S1. Detection of HCV PCR amplicons in the 5'-UTR, NS5B region, and C region using agarose gel electrophoresis.**

**
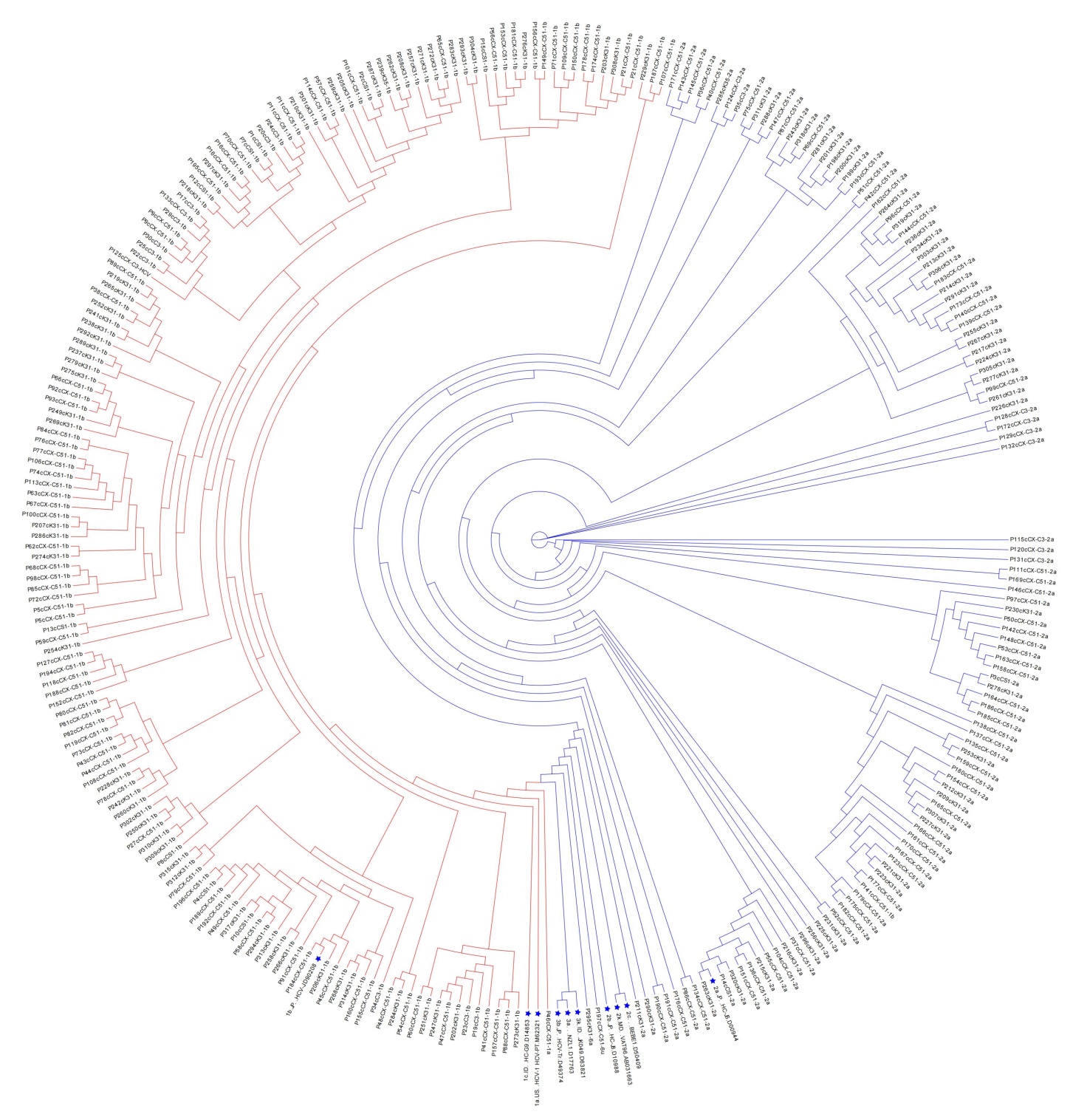
**

**Figure S2. Phylogenetic Tree Analysis of Core Region Nucleotide Sequences.**

Note: Sequences marked with an asterisk represent reference strain sequences; the branch lengths are proportional to the evolutionary distance and scale between sequences.

**
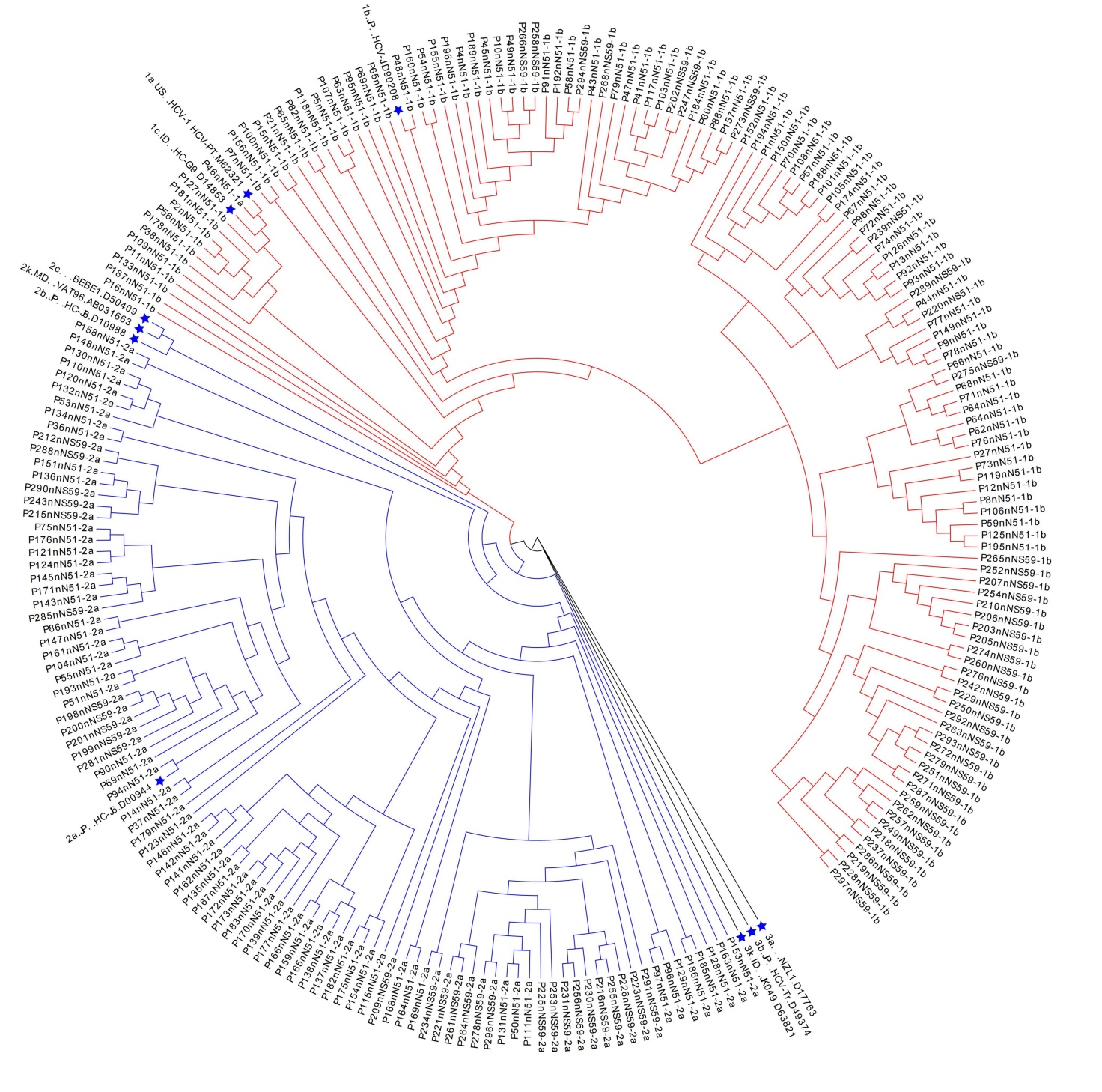
**

**Figure S3. Phylogenetic Tree Analysis of NS5B Region Nucleotide Sequences**

Note: Sequences marked with an asterisk represent reference strain sequences; the branch lengths are proportional to the evolutionary distance and scale between sequences.
